# Ultrasound-Guided Right Internal Jugular Vein Cannulation by Operators of Different Experience: A Randomized, Pilot Study

**DOI:** 10.7759/cureus.24381

**Published:** 2022-04-22

**Authors:** Sourav Nandy, Manas P Borthakur, Mohd Yunus, Habib Md R Karim, Samarjit Dey, Prithwis Bhattacharyya

**Affiliations:** 1 Anaesthesiology and Critical Care, Military Hospital Shillong, Shillong, IND; 2 Anaesthesiology and Critical Care, Guwahati Neurological Research Centre (GNRC) Hospital Dispur, Guwahati, IND; 3 Emergency Medicine and Trauma, All India Institute of Medical Sciences, Bhopal, Bhopal, IND; 4 Anaesthesia/Critical Care/Emergency Medicine, All India Institute of Medical Sciences, Raipur, Raipur, IND; 5 Anaesthesiology, All India Institute of Medical Sciences, Raipur, Raipur, IND; 6 Anaesthesiology and Critical Care, North Eastern Indira Gandhi Regional Institute of Health and Medical Sciences (NEIGRIHMS), Shillong, IND

**Keywords:** skill, experience, complications, ultrasonography, central venous cannulation

## Abstract

Background and aim: Currently, ultrasound-guided (US-guided) internal jugular vein (IJV) cannulation is the recommended technique. However, it has a learning curve and might be unsafe in inexperienced hands. The present study aimed to compare the performance and complications with two levels of experience in performing US-guided right IJV cannulation.

Methods: With informed consent, 108 procedures were performed after random allocation into two groups based on operator experience. An operator with experience in performing 30 or more ultrasound-guided IJV cannulation was considered an expert. The rate of successful cannulation, the time needed, number of attempts, and complication rate were measured. Quantitative continuous variables were compared using the unpaired student's t-test, and the chi-square test or Fisher's-exact test was used for the comparison of qualitative variables; P-value < 0.05 was considered significant.

Results: The successful cannulation rates were 100% versus 94.44% in the expert and non-expert groups, respectively; (P=0.0803). The mean time for successful cannulation and the percentage of patients who required ≥ two attempts were significantly lower in the expert group (33.28 seconds and 12.96% versus 95.42 seconds and 61.12%). Although the incidence of carotid artery puncture and hematoma (7.4% and 5.56%) was higher in the non-expert group, it was not statistically different; (P=1.00).

Conclusion: US-guided right IJV cannulation has a learning curve, and procedures as many as 30 US-guided IJV cannulation need to be observed and performed under the guidance to achieve it.

## Introduction

Central venous access is required in cardiothoracic surgeries for hemodynamic monitoring, potent vasoactive agents, irritant agents, heparin and protamine administration, etc. Among the sites used, the right internal jugular vein (IJV) approach is more preferred because of its easy accessibility, straight course, and relatively lower risk of pneumothorax [1,2].

Traditionally, IJV catheterization has been performed using the anatomical landmark technique utilizing its relations to the sternocleidomastoid muscle, clavicle, and internal carotid artery. The IJV runs anterolateral to the internal carotid artery and ends behind the inner side of the clavicular head of the sternocleidomastoid muscle near the medial end of the clavicle to join the subclavian vein. However, anatomical variations are also not uncommon [3].

Although the anatomical landmark technique is validated and time-tested, it is associated with many complications. A previous study using the landmark method reported successful IJV cannulation between 82% and 94.4% [4-6], and the rates of mechanical complications like carotid artery puncture were 7.7% to 20% [4-7], hematoma in 8.4%, and pneumothorax 2.4% to 5.8% [4-6]. However, these studies also reported that while using the US-guided method, the success rate of IJV cannulation was 100%, and the incidence of mechanical complications was significantly reduced [4-6].

Meta-analyses comparing the USG technique to landmark guided techniques concluded that fewer failures and fewer attempts were needed in USG techniques [8,9]. The National Institute for Health and Care Excellence (NICE) recommends USG for all elective central venous cannulations [10]. The Agency for Health Care Research and Quality of the United States has recommended USG-guided IJV cannulation as the best practice to improve patient safety [11]. However, the Centre for Disease Control (CDC) recommends a US-guided technique and only be used by fully trained clinicians for benefits [12], as this technique has a well-recognized learning curve.

Randomized studies analyzing the effects of the operator's experience on the effectiveness and incidence of complications when performing USG central venous catheterization are limited. The present study was designed to assess the effect of operators with two different experience levels.

## Materials and methods

This prospective, randomized, pilot study was carried out in a tertiary care teaching institute in northeastern India between January 2016 and February 2017. After approval from the institutional ethical committee and informed consent from the participants, patients aged between 10 and 60 years undergoing cardiothoracic surgery with the American Society of Anesthesiologists (ASA) physical status class II to IV were included in the study. Patients with bleeding disorders or anatomical deformity or infection, having a history of previous right IJV cannulation in the last one month, and burns at the insertion site were excluded, and so were patients with positive status for HIV, Hep-B, or Hep-C.

A total of 108 patients were assessed for the eligibility criteria; they satisfied the criteria, consented to, and were enrolled in the study. Computer-generated random numbers were placed inside closed envelopes, opened just before the randomization and allocation concealment procedure. They were allocated into two groups, an expert (N=54) and a non-expert (N=54). In the expert group, cannulation was done by a physician with experience of more than 30 USG IJV cannulation, and in the non-expert group, the operator had performed less than 30 USG IJV cannulation.

Patients of both the groups were pre-medicated with IV morphine 0.1mg/kg under oxygen supplementation and shifted to the operation theatre where ASA standard monitors were connected. Right radial artery cannulation was done and transduced for invasive blood pressure monitoring. General Anaesthesia (GA) was induced following standard institutional protocol. The trachea was intubated using an appropriate endotracheal tube facilitated with muscle relaxants; put on a mechanical ventilator attached to Mindray A7 (Mindray medical International Co, Ltd, Shenzhen, China) anesthesia workstation. Patients were then positioned in a 15-degree Trendelenburg position with a rolled towel under the shoulders and heads turned to the left side (approximate 45 degrees). The neck area was prepped and draped as per standard institutional practice. A 7.5-MHz linear ultrasound transducer was covered with ultrasonic gel, wrapped with a sterile Tegaderm® patch (3M India Ltd, Bengaluru), and connected to a real-time ultrasound unit Esaote (Genoa, Italy).

The skin was then dampened with sterile saline solution, and the transducer was placed in the groove near the apex of the triangle formed by the sternal and clavicular heads of the sternocleidomastoid muscle. The IJV was located with an 18G long needle guided by the ultrasound probe using the short axis view. When the needle appeared to be inside the IJV, as visualized in the ultrasound and also by the return of venous blood into the syringe, the Certofix® Trio (B. Braun Melsungen AG) central venous catheter was slid over the guidewire following Seldinger's technique.

Patient clinic demographics include age, sex, weight, height, body mass index (BMI), neck circumference at the thyroid cartilage level, and neck length (defined as the distance between the spinous process of the seventh cervical vertebra and occipital protuberance) were noted.

To objectively assess the time required for the procedure, instead of recording the procedure's total duration, only “access time” was recorded for each procedure. The “access time” was defined as the time between skin penetration and venous blood aspiration into the syringe. The only time interval between first skin penetration to the successful venous puncture was recorded in case of multiple puncture attempts.

The number of attempts required for each cannulation and complication (skin hematoma, carotid artery puncture, pneumothorax, hemothorax, brachial plexus irritation, or injury) was recorded. Carotid artery puncture was noted as pulsatile, with bright red blood in the syringe, and a postoperative chest x-ray diagnosed hemothorax or pneumothorax.

The literature is indeterminant concerning the experienced operator; we planned this study as a pilot project. Our institutional pre-study observation of 10 cases revealed a first pass success rate of 90% among the experts. We hypothesized that the non-expert group would have 75% of the experts' first success rate (i.e., 90*75%=67.5). We calculated the sample for this pilot study two-sided significance level (1-alpha) of 95% and Power (1-beta) of 80%, which gave us a sample of 51 per group by Fleiss methods. Five percent drop-out was added, and the final sample of 54 per group was planned. The sample size was calculated using open-source epidemiologic statistics for public health (www.openepi.com).

Demographic data, procedure, and complication-related data were entered in a Microsoft excel master chart, and incidences were calculated and expressed as an absolute number and percentage scale. Further statistical analysis was done using Statistical Package for Social Sciences (SPSS), Version 20.0 (IBM Corp., Armonk, NY). The Chi-square test or Fisher's-exact test (when any of the categories had a value of less than 5) was used for comparison of qualitative variables such as gender, number of attempts, success rate, and incidence of complications. Quantitative variables between the groups, i.e., mean age, mean weight, mean height, mean BMI, and mean duration of the procedure, were compared using an unpaired student's t-test.

## Results

A total of 108 procedures were performed during the study, 54 by the “Expert” (E) group and an equal number by the “Non-expert” (NE) group. None of the procedures had missing data or were excluded from the analysis. There was no statistically significant difference between the groups in terms of gender, mean age, height, mean weight, mean BMI, Mean neck length, and mean neck circumference (Table 1).

**Table 1:** Patients characteristics of the groups expressed in number, percentage#, and mean + standard deviation. BMI- body mass index, N - total number, cm - centimeter, kg - kilogram, # Fisher’s exact test

Characteristics	Expert Group [N=54]	Non-Expert Group [N=54]	P-value
Male^#^	32 (59.25)	26 (48.14)	0.2492
Female^#^	22 (40.74)	28 (51.85)	
Age in years	38.78±14.77	37.33±14.26	0.6048
Weight in kg	52.30±12.24	51.31±12.25	0.6753
Height in cm	1.55±0.13	1.52±0.14	0.2511
BMI in kg/m^2^	21.51±3.15	21.86±3.46	0.5837
Neck length in Male (cm)	12.66±0.90	12.38±1.30	0.337
Neck length in Female (cm)	12.68±0.82	12.75±1.21	0.817
Neck circumference Male (cm)	33.37±5.87	33.88±4.88	0.724
Neck circumference in Female (cm)	32.95±4.84	32.57±5.25	0.794

The mean time (Table 2) for successful cannulation was significantly lower in the Expert Group (33.28±7.47 seconds) than in the Non-expert group (95.42±89.72). Even though the overall success rate (Table 2) was higher in the Expert group (100%) than in the Non-expert group (94.44 %), this was not statistically significant. The percentage of patients in whom ≥ two attempts were made before successful cannulation was significantly higher in the Non-expert group than in the Expert group. Although the incidence of carotid artery puncture and hematoma (Table 2) (7.4% and 5.56%), respectively, was higher in the Non-Expert group, it was not significantly different from that in the Expert group (1.85% and 1.85%). Complications (Table 2) like pneumothorax, haemothorax, brachial plexus irritation, or injury did not occur in both groups.

**Table 2:** Outcome variables in expert and non-expert groups compared using Fisher's exact test. IJV - internal jugular vein, N - total number. The time is presented as mean ± standard deviation.

Characteristics	Expert Group [N = 54]	Non-expert Group [N = 54]	p-value
Time for successful IJV cannulation(sec)	33.28 ± 7.47	95.42 ± 89.72	<0.0001
Number of attempts - One	47 (87.03%)	21 (38.88%)	< 0.0001
Number of attempts - Two	7 (12.96%)	22 (40.74%)	0.0012
Number of attempts - Three	0	7 (12.96%)	0.0065
Number of attempts - Four or more	0	4 (7.4%)	0.0426
Overall success rate	54 (100%)	51 (94.44%)	0.0803
Complications - No Complication	52 (96.29%)	47 (87.03%)	0.605
Hematoma	1 (1.85%)	3 (5.56%)	0.3097
Carotid artery Puncture	1 (1.85%)	4 (7.4%)	0.1717

## Discussion

The present randomized, single-blind, pilot study aimed to compare the effect of the operator's experience on the efficacy and the incidence of complications in USG IJV cannulation. Our study findings will help determine the USG IJV cannulation's learning curve, provide a training protocol for trainees and residents, and improve patient safety. NICE's position paper on the guidance on the US use for locating vein and cannulation indicates the deficiency and inconsistency in the term used for experts [13]. With the advancement of technology and the relatively well availability of US machines in most operation theatre and critical care units, it is essential to know the rationale, effectiveness, and safe use of the device on patients. Although non-invasive, familiarization with the technique and technology is crucial. To execute USG central venous cannulations, the user should have a sound knowledge of the sono-anatomy and should be able to interpret the 2D images of vessels and the surrounding. To develop hand-eye coordination for optimal probe and needle placement in the 3-dimensional (3D) plane, the user should have considerable practice and experience. Therefore, it is prudent to demarcate the required experience (procedures needed to be observed and mentored) before the trainees/residents are given free-hand.

In the present study, the successful cannulation rate was 100% in the Expert Group and 94.44% in the Non Expert group, which gives an overall success rate of 97.22%, consistent with the recent Cochrane systematic reviews and meta-analyses data [14]. In the present study, only 38.88% of cannulations in the Non-Expert group were accomplished in the first attempt, which was significantly lower than the Expert group (87.03%). Palepu et al. [15] reported that 84.4% of IJV cannulations were accomplished in the first attempt when done under USG guidance, and Bansal et al. [16] reported a success rate of 86.7% for the same. These values are similar to the first attempt success rate of the Expert group in this study. In the present study, the percentage of patients who required ≥ two attempts were significantly more in the non-expert group (61.12%) than in the Expert group (12.96%), demonstrating that the operator's experience is essential in determining the success of USG guided IV calculations. Previous studies have found that the meantime for successful cannulation using a US-guided technique was 17.1 ± 16.5, 646, and 77±108 seconds, respectively [4,17]. In the present study, the time required for successful IJV puncture in the expert group was 33.28±7.47 seconds, which agrees with the literature; however, in the non-expert group, the time required for successful IJV puncture was significantly higher, 95.42±89.72. It was similar to the puncture time reported in the Turker et al. study [18]. Augoustides et al. in a non-randomized observational, prospective study conducted in a university hospital, showed that the rate of complications like carotid artery puncture during USG-guided IJV cannulation depends on the experience and exposure of the operator [19]. The arterial puncture rate was lower in the senior operators than the junior operators, but the difference was not statistically significant. Like these observations, in the present study, the rate of complications like carotid artery puncture and hematoma was lower in the Expert Group (1.85% and 1.85%, respectively) compared to the non-expert group (7.4% and 5.56%, respectively). However, the difference was not statistically significant (P-value 0.3097 and 0.1717, respectively). However, it was noted that in all the cases of carotid artery puncture that occurred in the Expert Group, there was hematoma formation, which suggests that those cases might have intrinsically difficult cannulation.

The present study gives an approximation of the numbers of US-guided right IJV cannulations that need to be observed and performed for the learning curve. Nevertheless, the division was arbitrary, which is a limitation. Therefore, future studies will be required with different experiences and multiple groups for better determination and finding the ideal numbers.

## Conclusions

The present single-center, single-blind, randomized, pilot study indicates that US-guided right IJV cannulation has a learning curve; as many as 30 cannulations need to be observed and performed under a US-guided technique to achieve competency. However, it is just an approximation, and the ideal number might be different. For precise demarcation between experts and non-experts, further studies will be required.
